# Benign gastro-colic fistula in a woman presenting with weight loss and intermittent vomiting: a case report

**DOI:** 10.1186/1752-1947-5-313

**Published:** 2011-07-14

**Authors:** Kate Barrett, Michael W Hii, Richard J Cade

**Affiliations:** 1 St Vincent's Hospital Melbourne, PO Box 2900 Fitzroy, Victoria 3065, Australia

## Abstract

**Introduction:**

Benign gastro-colic fistula is a rare occurrence in modern surgery due to the progress in medical management of gastric ulcer disease. Here we report the first case of benign gastro-colic fistula occurring whilst on proton-pump inhibitor therapy. This is a case study of benign gastro-colic fistula and review of the available literature in regards to etiology, diagnosis, management and prognosis.

**Case presentation:**

An 84-year-old woman of Caucasian background presented with 12 months of worsening abdominal pain, nausea, vomiting, diarrhea and weight loss on a background of known gastric ulcer disease.

**Conclusion:**

The leading cause of gastro-colic fistulae has changed from benign to malignant due to improved medical management of gastric ulcer disease. The rarity and non-specific symptoms of gastro-colic fistula make the diagnosis difficult and it is best made by barium enema; however, computed tomography has not been formally evaluated. Surgical management with *en bloc *resection of the fistula tract is the preferred treatment. Benign gastro-colic fistulae are becoming exceedingly rare in the context of modern medical management of gastric ulcer disease. Surgical management is the gold standard for both benign and malignant disease.

## Introduction

Gastro-colic fistulae are described as presenting with the clinical triad of diarrhea, nausea/vomiting and weight loss [1]. However, all three features are said to occur in only 30% of patients. Other symptoms include malnutrition with cachexia, anemia, abdominal pain and fecal halitosis that is present in over 50% of patients [1,2].

Malignant gastro-colic fistulae were first described in 1755 by Haller [3]. Gastro-colic fistulae due to benign peptic ulcer disease were described by Firth in 1920 [4]. Gastrointestinal malignant disease is the predominant cause today: colonic adenocarcinoma in the Western world, gastric carcinoma predominating in Japan [2,5]. Other malignant causes include gastric lymphoma, carcinoid tumors of the colon and locally invasive malignant tumors of the biliary tree, pancreas and duodenum [1]. Benign causes described include peptic ulcer, gastric tuberculosis, trauma, syphilis, retroperitoneal sarcoma, Crohn's disease and pancreatitis [2,3].

The overall incidence of gastro-colic fistula has decreased since the advent of effective medical management of gastric ulcer disease. Post-surgical-resection-associated fistulae and fistulae related to the use of non-steroidal anti-inflammatory medications were the most reported causes of benign gastro-colic fistulae [2,4,6]. In a single case series from 1955, prior to the advent of H2 antagonists and proton pump inhibitors, it was reported that up to 10% of patients post-gastrectomy for benign gastric ulcer subsequently developed a gastro-colic fistula [7]. Fistulae in gastric ulcer disease in the setting of proton pump inhibitor use are exceedingly rare and to the best of our knowledge this is the first documented case.

A barium enema is the radiological modality of choice for diagnosis of gastro-colic fistulae, with specificity of 90-100% compared with a barium meal that has a false negative rate of 30-70% [1,3]. Endoscopic investigations are recommended to exclude malignant disease. Computed tomography (CT) has not been evaluated for sensitivity and specificity but has been reported in one case series as a useful adjunct in diagnosis and staging.

The treatment of choice for a gastro-colic fistula is *en bloc *surgical resection of the fistula tract with a margin of adjacent tissue [1,3,4,8]. This allows disease free margins in malignant disease and decreases the recurrence rate in benign disease, which has been reported to be up to 12%. The recurrence rate is higher if simple excision of the fistula tract is used for initial management [1].

Several cases of medical or minimally invasive management of gastro-colic fistulae have been described and may be suitable where malignant disease has been excluded and/or surgical intervention is not appropriate. Endoscopic injection of the fistula tract with fibrin has shown to be effective in several case reports [1].

Prognosis for gastro-colic fistula has been thought to be quite poor. Between 1963 and 1994, the longest recorded survival post-resection for gastro-colic fistula due to malignant disease was nine to ten years [1,5]. Post-operative mortality has been reported to be as high as 25%, presumably due to co-morbidity and de-conditioning of the patients [1].

One case series of six patients reported one post-operative death due to underlying co-morbid conditions. The remaining cases were followed for a mean of 66 months, with one further death due to an unrelated underlying co-morbid condition [1]. However, there have been very few recent studies and advances in surgical techniques and post-operative care as well as nutritional optimization suggest empirically that prognosis may have improved.

## Case presentation

An 84-year-old Caucasian woman presented for repeat gastroscopy for follow-up of a benign gastric ulcer. She gave a 12-month history of worsening abdominal pain, nausea, non-feculent vomiting, diarrhea and approximately 20 kilogram weight loss. She denied any hematemesis, melena or fever. At presentation our patient was frail and emaciated. Regarding clinical examination, there were no abnormal abdominal findings.

A chronic gastric ulcer on the greater curve of her stomach had been first diagnosed at gastroscopy eighteen months earlier. Since then she had undergone four further gastroscopies without any change. Biopsies had only demonstrated features of chronic inflammatory change. *Helicobacter pylori *had never been identified. Our patient was taking aspirin for cardiovascular prophylaxis and had been started on pantoprazole at 40 milligrams twice daily when the ulcer was first identified. Our patient's general practitioner confirmed prescription requests for this medication.

**Figure 1:**
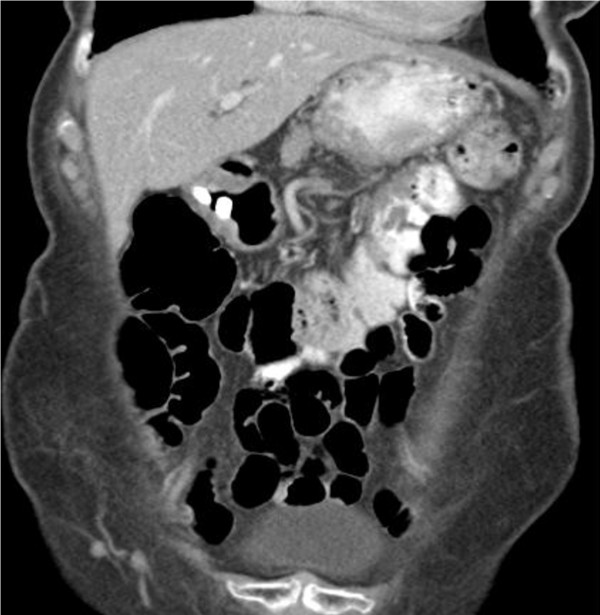
**Coronal CT scan post-gastroscopy revealing gastro-colic fistula demonstrated by oral contrast in the stomach and distal transverse colon and absence of contrast in the duodenum**. There are associated inflammatory changes around the transverse colon.

On this occasion, gastroscopy revealed a deep ulcer of the greater curve of the stomach that appeared to penetrate the muscular layer and was highly suspicious of a fistula. The pathological report of the performed biopsy showed chronic inflammatory changes. An abdominal CT demonstrated a fistula between the stomach and transverse colon and excluded malignant disease. Contrast CT successfully diagnosed a fistula, excluded locally invasive disease and allowed pre-operative planning in a single step. A colonoscopy showed no evidence of primary colonic disease and failed to visualize the fistulous opening (Figure 1).

**Figure 2:**
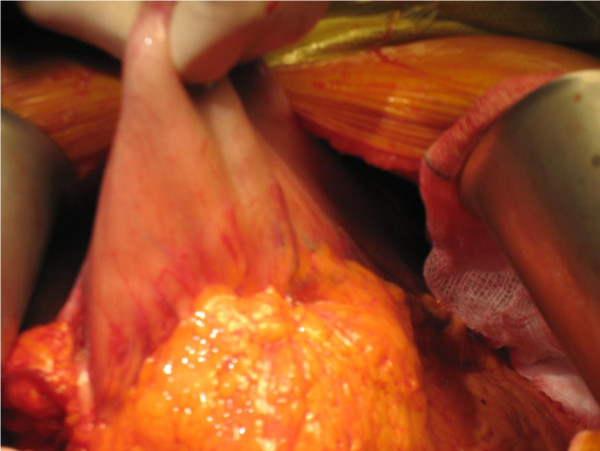
**The operative field showing the stomach attached to omentum and transverse colon**.

**Figure 3:**
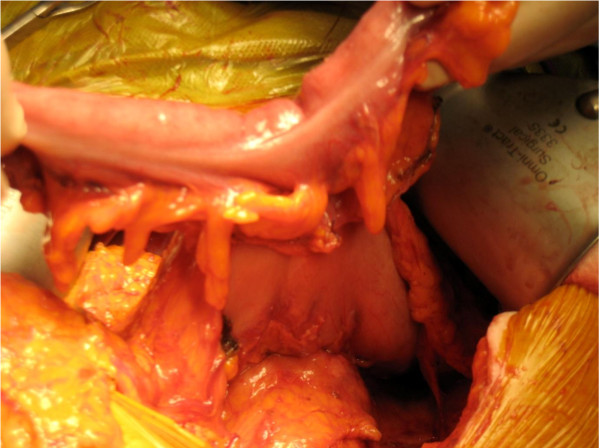
**The operative field demonstrating the stomach attached to the transverse colon**.

**Figure 4:**
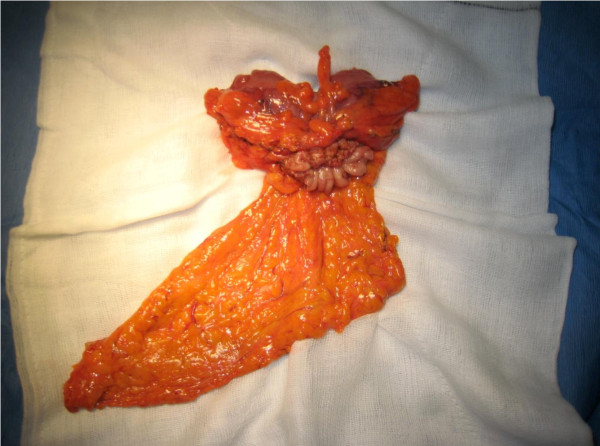
***En bloc *surgical resection of the distal stomach, transverse colon and surrounding inflammatory tissue**.

At laparotomy there were dense adhesions between the greater curve of the stomach and the distal transverse colon. The gastric ulcer together with the fistulous track and colonic opening were excised *en bloc *and primary anastomoses performed as malignant disease could not be definitely ruled out. A feeding jejunostomy was performed (Figures 2, 3, 4). Histopathology showed chronic inflammatory changes consistent with gastric ulceration. No malignancy was identified.

Our patient was discharged to a peripheral hospital on the twentieth post-operative day tolerating an oral diet.

## Conclusion

A gastro-colic fistula commonly presents with non-specific symptoms of diarrhea, nausea and vomiting and weight loss, thus making it a difficult diagnosis. The rarity of this condition, and alteration in the underlying etiology due to the advent of medical management of gastric ulcer disease, make benign gastro-colic fistula a very rare diagnosis. This case is important as it highlights the non-specific presentation of the disorder and is the first case documented in which benign gastric ulcer disease treated with proton-pump inhibitors progressed to gastro-colic fistula.

## Consent

Written informed consent was obtained from the patient for publication of this case report and any accompanying images. A copy of the written consent is available for review by the Editor-in-Chief of this journal.

## Competing interests

The authors declare that they have no competing interests.

## Authors' contributions

MH and RC were the surgeons involved in the care of the patient. KB researched the background management of the patient and performed the literature review. All authors read and approved the final manuscript.
